# Left atrial remodelling following transcatheter aortic valve implantation (TAVI) and surgical aortic valve replacement (SAVR)

**DOI:** 10.1186/1532-429X-17-S1-P182

**Published:** 2015-02-03

**Authors:** Tarique A Musa, Laura E Dobson, Akhlaque Uddin, Timothy A Fairbairn, Ananth Kidambi, David P Ripley, Peter P Swoboda, Bara Erhayiem, Pankaj Garg, Adam K McDiarmid, Sven Plein, John P Greenwood

**Affiliations:** Multidisciplinary Cardiovascular Research Centre & Leeds Institute for Cardiovascular and Metabolic Medicine, University of Leeds, Leeds, UK

## Background

Left atrial (LA) size is a marker of poor prognosis in a variety of cardiovascular conditions. Severe aortic stenosis results in elevated LV afterload with compensatory hypertrophy and increased filling pressures. This is associated with progressive left atrial enlargement and dysfunction. Intervention for aortic stenosis results in LV reverse remodelling, however the effect of TAVI or SAVR upon LA function remains poorly understood and the two treatments have not been directly compared.

We sought to accurately determine LA size and function using CMR in patients with severe symptomatic aortic stenosis and identify changes following TAVI compared to SAVR.

## Methods

All patients underwent an identical 1.5T CMR protocol (Intera, Phillips Healthcare, Best, The Netherlands). Multi-slice, multi-phase cine imaging was performed using a SSFP pulse sequence in the short axis (8 mm thickness, 0 mm gap, 30 phases, typical field of view (FOV) 340 mm) to cover the entire left and right ventricles. All patients were in sinus rhythm at time of CMR imaging; patients with atrial fibrillation were excluded. Offline analysis (CVI42, Circle Cardiovascular Imaging, Calgary, Alberta, Canada) involved measuring the maximum (end systole before mitral valve opening; LAV_max_) and minimum (end diastole right after mitral valve closure; LAV_min_) LA volumes using the biplane area-length method from the apical 4-and 2-chamber cines. Subsequently the total LA emptying fraction was derived as: (LAVmax -LAVmin)*100/LAVmax.

## Results

23 SAVR patients (age 72.7±7.5 years, 83% male, EuroSCORE II 1.40±1.11%) and 23 TAVI patients (age 80.7±6.9 years, 57% male, EuroSCORE II 4.99±2.97%) were studied before and 6 months following valve replacement. The left atria of the TAVI group were significantly more dilated at baseline than those of the SAVR group (p=0.039) however both groups were comparable at 6 months (p=0.227). Similarly, the LA emptying fraction of the TAVI group was significantly lower than the SAVR group at baseline (p=0.003) with comparable function seen at 6 months between the groups (p=0.08).

## Conclusions

TAVI, but not SAVR, was associated with a significant reduction in LA volume and concomitant improvement in emptying fraction at 6 months. These preliminary findings may reflect worse LA function at baseline in the TAVI group or improved valvular haemodynamics with TAVI compared to SAVR.

## Funding

This study was part-funded by the British Heart Foundation (BHF) (PG/11/126/29321).

**Table 1:** LA remodelling following TAVI and SAVR

TAVI	Baseline	6 months	p Value
Maximum LA volume (mls/m2)	63.3 ± 17.0	52.8 ± 14.0	0.001
Total LA emptying fraction (%)	36.9 ± 12.6	43.4 ± 10.4	0.011
SAVR	Baseline	6 months	p Value
Maximum LA volume (mls/m2)	53.2 ± 15.1	48.2 ± 11.7	0.09
Total LA emptying fraction (%)	48.5 ± 12.8	48.7 ± 9.1	0.945

